# Cytokine cascade and networks among MSM HIV seroconverters: implications for early immunotherapy

**DOI:** 10.1038/srep36234

**Published:** 2016-11-10

**Authors:** Xiaojie Huang, Xinchao Liu, Kathrine Meyers, Lihong Liu, Bin Su, Pengfei Wang, Zhen Li, Lan Li, Tong Zhang, Ning Li, Hui Chen, Haiying Li, Hao Wu

**Affiliations:** 1 Center for Infectious Diseases, Beijing You’an Hospital, Capital Medical University, Beijing 100069, China; 2 Infectious Diseases Department, Peking Union Medical College Hospital, Beijing, 100730, China; 3 The Aaron Diamond AIDS Research Center, New York, NY 10016, United States; 4 School of Biomedical Engineering, Capital Medical University, Beijing 100069, China

## Abstract

The timing, intensity and duration of the cytokine cascade and reorganized interrelations in cytokine networks are not fully understood during acute HIV-1 infection (AHI). Using sequential plasma samples collected over three years post-infection in a cohort of MSM HIV-1 seroconvertors, we determined the early kinetics of cytokine levels during FiebigI-IV stages using Luminex-based multiplex assays. Cytokines were quantified and relationships between cytokines were assessed by Spearman correlation. Compared with HIV-negative MSM, HIV-infected individuals had significantly increased multiple plasma cytokines, including GM-CSF, IFN-α2, IL-12p70, IP-10 and VEGF, during both acute and chronic stages of infection. Furthermore, rapid disease progressors (RDPs) had earlier and more robust cytokine storms, compared with slow disease progressors (SDPs) (49.6 days vs. 74.9 days, respectively; 6.7-fold vs. 3.7-fold change of cytokines, respectively), suggesting the faster and stronger cytokine storm during AHI could promote disease progression. On the other hand, HIV-1 infection induced more interlocked cytokines network, establishing new strong correlations and imposing a higher rigidity. There were, respectively, 146 (44.9%) statistically significant correlations of cytokines in RDPs and 241 (74.2%) in SDPs (*p* < 0.001). This study suggests that immunomodulatory interventions aimed at controlling cytokine storm in AHI may be beneficial to slow eventual disease progression.

When the immune system is fighting pathogens, cytokines activate immune cells such as T cells and macrophages, stimulating them to produce more cytokines resulting in so-called cytokine storms or cascades^1^,^2^. There is growing evidence that the immune responses initiated during acute HIV infection can play critical roles in modulating of disease progression. Several studies investigated the cascade of cytokine production in the periphery and show an initial rapid production of IFN-α, followed by tumor necrosis factor (TNF)-α, inducible protein (IP)-10, and interleukin (IL)-18, IL-10 and IFN-γ production, while others have not observed these changes or have reported opposing findings^3^,^4^,^5^,^6^,^7^,^8^. The discrepancies likely reflect the diversity and unity of dynamics during AHI but also could be due in part to the variation in the timing of sample collection among studies. Some studies were cross-sectional, while others focused on time points relatively late in AHI^9^,^10^. The cytokine storms during AHI can act as a double-edged sword, as they have the potential to cause significant damage to virus-specific immunity but also inhibit infection by reducing T cell recruitment and activation^11^. Some studies show that type I-IFN plays a role in slowing disease progression by inducing multiple antiviral genes and limiting initial viral replication, while others have reported that type I-IFN signaling is linked to immune activation and viral persistence and blocking of type I-IFN during LCMV infection enhances viral clearance^12^,^13^,^14^. These contradictory reports suggest that there is a need to investigate the dynamic of cytokines in natural HIV-1 infection.

HIV-1 infection does not only increase cytokine levels, but also reorganizes cytokine networks, establishes new strong correlations between various cytokines and thus imposes a high rigidity to the cytokine network^15^,^16^. We speculate that the “cytokine cascade” and “new order” in this network may be one of the important factors determining HIV-1-mediated immunodeficiency. However, the very early kinetics of cytokines during the first weeks of infection is unknown^17^,^18^,^19^. To address this deficit, we took advantage of a well-established longitudinal acute HIV-1 cohort (Beijing PRIMO cohort study) to investigate the dynamics of 26 cytokines in plasma from pre-infection to acute and chronic phases of infection^20^,^21^.

## Results

### Basic characteristics of the study participants

Twenty acute HIV-infected individuals from an MSM cohort were recruited into this study^22^. Basic information about 10 rapid disease progressors (RDPs) and 10 slow disease progressors (SDPs) is described in Table 1.

### Multiple cytokine storms during acute phase of HIV-1 infection

**Figure 1:**
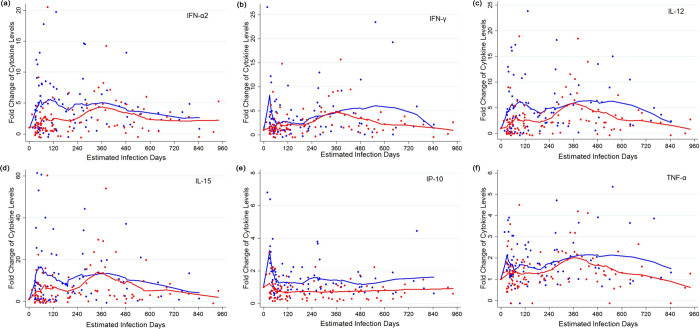
The dynamic fold changes of plasma (**a**) IFN-α2, (**b**) IFN-γ, (**c**) IL-12, (**d**) IL-15, (**e**) IP-10 and (**f**) TNF-α in rapid disease progessors (RDPs) (blue dots) and slow disease progressors (SDPs) (red dots). The blue and red lines are the locally weighted scatterplot smoothing curves for RDPs and SDPs, respectively.

In order to investigate the dynamics of plasma cytokines during HIV infection, sequential longitudinal plasma samples collected from pre-infection to over three years post-infection were analyzed. As shown in Table 2, compared with HIV-negative MSM controls, HIV-infected individuals had significantly increased plasma cytokines between 180 days and 3 years post-infection. Increased cytokines included GM-CSF, IFN-α2, IL-12p70, IP-10 and VEGF (*p* < 0.001), and much higher levels were observed in RDPs compared to those in SDPs. Interestingly, as shown in Fig. 1 (IFN-α2, IFN-γ, IL-12, IL-15, IP-10 and TNF-α as a representative) and S1 (other 20 cytokines), during acute stage of infection, plasma cytokines FGF-2, GM-CSF, IFN-α2, IFN-γ, IL-1β, IL-1ra, IL-2, IL-6, IL-10, IP-10, IL-12-p70, TNF-α and VEGF had more positive changes in RDPs than those in SDP.

**Figure 2:**
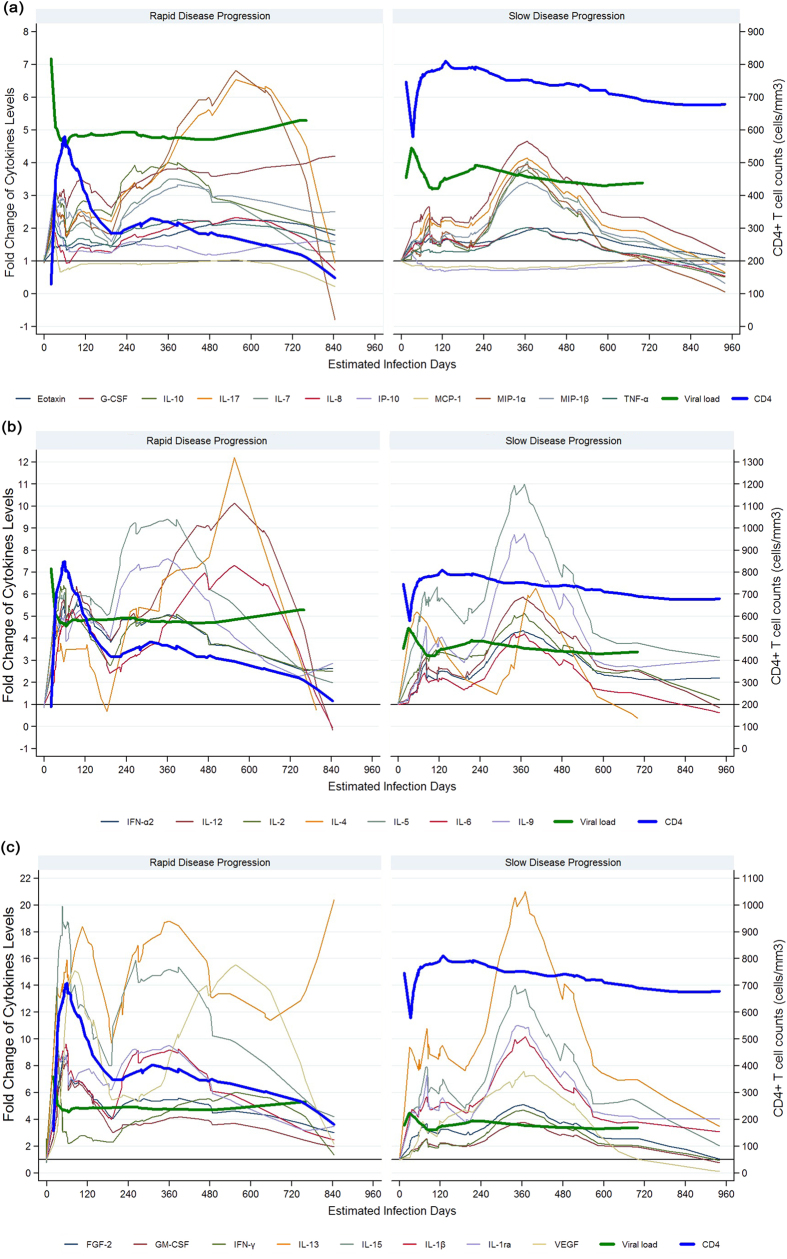
Successive waves of 26 cytokines, viral load and CD4^+^ T cell counts in HIV-1-infected individuals in rapid (left) and slow (right) progression groups. The solid lines of cytokines were locally weighted scatterplot smoothing curves (LOWESS) fitted on fold changes of each cytokine for all rapid or slow disease progressors, respectively. Viral load (log copies/mL, thick blue line) is also plotted on left Y axis. (**a**) 11 cytokines (Eotaxin, G-CSF, IL-7, IL-8, IL-10, IL-17, IP-10, MCP-1, MIP-1α, MIP-1β and TNF-α) with level increased less than 7 fold. (**b**) 7 cytokines (IFN-a2, IL-2, IL-4, IL-5, IL-6, IL-8 and IL-12) with level increased between 7- and 12-fold. (**c**) 8 cytokines (FGF-2, GM-CSF, IFN-γ, IL-13, IL-15, IL-1β, IL-1ra and VEGF) with level increased more than 12-fold.

Considering that the dynamic changes in cytokines might be related to disease progression, we analyzed plasma levels of each cytokine and the time to reach peak value. We found that some cytokines rapidly increased to their highest levels, whereas others took much longer to do so (Table 3). For example, plasma IP-10, IL-8, and MCP-1 in RDPs reached peak value at 30 days post infection, whereas IL-6, GM-CSF, and VEGF were delayed after 60 days post infection in RDPs. More interestingly, RDPs had much earlier and stronger cytokine storms in acute stage, compared with SDPs (49.6 days vs. 74.9 days, respectively; 6.7-fold vs. 3.7-fold change, respectively). For example, a 13.7-fold increase in IFN-γ was seen in RDP at day 30 after infection, but only a 2.5-fold increase in SDP at day 81 after infection. 19 of 26 cytokines in RDPs had an approximate time of initial elevation to peak value between 30 and 60 days post-infection, whereas only 3 of 26 cytokines in SDPs did so. Additionally, the hierarchy of cytokine induction differed between SDP and RDP groups. RDPs had significantly and early increased IFN-α, TNF-α, IL-1β, IL-1ra, but a delayed IL-13 and VEGF compared with SDPs. Surprisingly, IL-13 in SDP reached peak value at day 33 after infection, compared to day 105 in RDP. Considering that cytokine storms are triggered by HIV, we further analyzed the dynamics of virus replication and its kinetic relationship with cytokine storms. To our surprise, as shown in Fig. 2, we found the drop of viral load from peak to set-point was followed by a decline of most of cytokines in RDPs, whereas cytokines increased in SDPs.

### Second wave of plasma cytokine storms during chronic stage of HIV infection

We next asked whether HIV infected individuals have higher sustained levels of plasma cytokines during chronic infection compared to HIV-negative controls. As shown in Table 2, compared with HIV-negative MSM, chronically HIV-infected individuals had significantly increased plasma GM-CSF, IFN-α2, IL-12p70, IP-10 and VEGF, and much higher levels were observed in RDPs compared to SDPs (*p* < 0.001). Then we asked whether RDPs had higher levels of plasma cytokines than SDPs in chronic infection as in acute disease. As shown in Fig. 2, both RDPs and SDPs had high levels of plasma cytokines after viral set point, and had a second wave of cytokines storms during chronic stages. FGF-2, GM-CSF, IFN-γ, IL-13, IL-15, IL-1β, IL-1ra and VEGF had increased more than 12-fold. 7 of 26 cytokines increased 7–12 fold, and 11 cytokines have less than 7-fold changes. Interesting, there is no significant difference on the levels and the time to reach peak value of the second wave between two groups (data not shown).

### Correlation among plasma cytokine concentrations during HIV-1 infection

**Figure 3:**
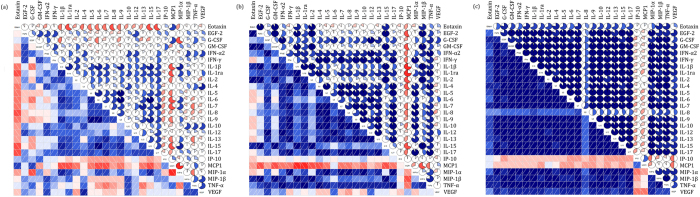
Correlograms of the correlations among 26 plasma cytokine concentrations for (**a**) healthy subjects, (**b**) HIV-infected individuals with rapid disease progression, and (**c**) HIV-infected individuals with slow disease progression. A blue and red color represent a positive and negative correlation between the two plasma cytokine concentrations that meet at that cell, respectively. The darker and more saturated the color, the greater the magnitude of the correlation.

HIV disease progression resulted in a significant modification of the interconnections between cytokines belonging to functionally distinct classes: the median correlation coefficients (0.890 vs. 0.524) were significantly different in SDPs and RDPs (*p* < 0.001), and they were both significantly different from plasma from HIV-uninfected (or healthy) subjects (0.186, *p* < 0.001) (Fig. 3). Furthermore, in RDPs, there were 146 (44.9%) statistically significant correlations between the levels of individual cytokines. In contrast, in SDPs, there were 241 (74.2%) such correlations (*p* < 0.001). Thus, the cytokine networks become more interlocked in SDPs than those in RDPs: 114 new correlations were established, and 19 correlations were lost. 97 pre-existing correlations increased in magnitude, 29 decreased, and 1 did not. For example, for IL-2, only correlations with IL-15, MCP-1, MIP-1β and TNF-α were found in RDPs, while 11 new statistically significant correlations, including those with IL-4 and IL-10, were established for this cytokine in SDPs. In another example, a relatively weak correlation of IL-6 with IL-10 in RDPs (r = 0.647, p < 0.001) became a very strong one in SDPs (r = 0.993, p < 0.001).

## Discussion

Some studies have previously shown that the cytokine cascade found in AHI might contribute to control of viral replication^2^,^23^. However, both the extent and duration of exponential cytokine expansion during acute infection are poorly understood^2^,^22^,^24^. Very few studies have been able to investigate the very early events during the first several weeks post infection, since the exact infection date is hard to know and the plasma samples in acute infection are difficult to be collected. This study reported the dynamic profile of cytokines from pre-infection to acute, and chronic stage of infection.

The first finding in this study was that RDPs had rapidly increased cytokines in peripheral blood in very early after infection, whereas SDP had delayed and only mild increases of plasma cytokines. These data overwhelming suggested that increased cytokines in very early infection were related to immunopathogenesis and rapid disease progression, which is consistent with other reports and findings in HBV, HCV and SARS (Severe Acute Respiratory Syndrome) infections^5^,^6^,^25^,^26^,^27^. Second, we found RDPs had a disparate cytokine profile compared with SDPs. Multiple cytokines in RDPs, including TNF-α, IL-8, IP-10, MCP-1, MIP-1α, IL-1ra, IL-10, G-CSF, IFN-γ, IL-4 and IL-17, reached peak value in 4 to 5 weeks after infection, whereas only IP-10 and IL-13 in SPDs did so, and lack of TNF-α in SDPs. Consistent with our results, another report had also shown elevations in IL-10, TNF-α, and IFN-γ in acute HIV infection^3^. IFN-γ is secreted by NK cells, Th1 cells and CD8^+^ cytotoxic T lymphocytes during active infection. IFN-γ has broad effects on immune activation, proinflammatory responses, and immune modulation^28^. Interesting, we found IL-13 in SDP reached peak value at much earlier time than RDPs. An *in vitro* study had shown that IL-13 decreased TNF-α secreting and modulated monocytes towards supporting Ag-specific cell medicated responses^29^. These data suggested that the rapid increased IL-13 in SDPs might play a role in augmenting Ag-specific cell medicated responses and be related to slow disease progression.

Consistent with other reports on “cytokine storms” during AHI^2^, we found an ordered sequence of increased cytokines during the acute stage in RDP. The first rapid and transient elevations in TNF-α, IFN-γ, IL-4, IL-8, G-CSF, and IP-10 were at 2 weeks after detection of peak viral load and declined in parallel with the decrease of viral replication, which suggested that the virus directly or indirectly drives the production of cytokine. Rapid and more-sustained elevations in IL-1ra, MIP-1α, IL-5, IL-10 and IL-17 levels were followed by IL-1β, IL-2, IL-7, IL-9, IL-12, IL-15, IFN-α2, MIP-1β, FGF-2 and GM-CSF at over 2 months post-infection, and accompanied by the recovery of CD4^+^ cells. A lately increased cytokine IL-6, VEGF and IL-13 were at around 3 months after infection. This complex change on the dynamic of cytokines in RDPs did not happen in SDPs, who had much delayed and milder changes in plasma cytokines. These data suggested that vigorous cytokines storm in RDPs very early after infection reflected the battle between virus replication and host immune response, and resulted in immunopathogenesis and rapid disease progression^30^,^31^.

It is widely accepted that cytokines form a coordinating complex network. This study allowed us to reveal the interaction between different cytokines. The production of an immunosuppressive cytokine like IL-10 became a very strong correlation of IL-6 in slow progression group, compared with rapid progression group. This is consistent with reports from Dr. Andrea Lisco’s group and others that have demonstrated that in the course of HIV infection various cytokines are up- or down-regulated in blood and semen, and are more interlocked than uninfected individuals^10^,^15^,^32^,^33^. Here we more precisely characterized the “rigidity” of the network in slow and rapid progressors and found cytokines were more related in SDPs than in RDPs. We revealed that HIV-1 infection imposes a qualitatively new order on the cytokine network and its underlying cellular networks, which may contribute to immunodeficiency. Although the multiple functions of many cytokines are not completely understood and we do not know the exact functional meaning of correlations and whether they have direct effects on the immune response, our study shows that many positive correlations are built in the blood of HIV-1-infected individuals. A larger cohort study may reveal critical factors associated with the regulation of the cytokines network and indicate novel targets for therapy strategies. There were some limitations in this study, including the small number of patients, which introduces the possibility of bias that could lead to an underestimation of the true differences. Additionally, there were more individuals with syphilis in RDP than SDP, which may contribute to immune activation and lead to transient increases in HIV-1 RNA plasma levels and decreases in CD4^+^ cell counts, however, it had been shown that syphilis has no apparent long-term impact on HIV-1 progression^34^.

In summary, to our knowledge this study is the first to investigate the cytokine cascade and associated networks among MSM HIV-1 seroconverters. In the study, we constructed a comprehensive picture of the dynamics of 26 cytokines in the earliest stage of infection by analyzing sequential plasma samples from acute HIV-infected MSM. Our study revealed an impressive and broad cytokine storm in AHI in patients with rapid disease progression, and suggested a rationale that controlling cytokine storms in very early infection (in the first 2 months) may be beneficial to immune recovery and slow disease progression.

## Methods

### Ethical Issues

The study protocol and all relevant experiments have been approved by the Beijing You’an Hospital Research Ethics Committee. All study participants provided written informed consent upon admission for their information to be stored and used for research. The methods were carried out in accordance with the approved guidelines and regulations.

### Study population and Design

Consenting MSM, newly infected with HIV-1 were recruited from the Beijing PRIMO Cohort, a prospective cohort of HIV-negative MSM who were screened for HIV every 8–12 weeks^20^. Estimated time of infection was defined as the mid-point between the last HIV antibody negative test and the first HIV antibody positive test, or as 14 days prior to a positive RNA PCR assay on the same day as a negative HIV Enzyme Immunoassay. Out of 450 acute cases detected between 2 to 6 weeks post infection, we selected 10 “rapid progressors” whose CD4^+^ T cells decreased to below 200 cell/μL within about 3 years, and 10 “slow progressors” who retained CD4+ T cells above 500 cell/μL at 3 years post-infection (all in the absence of treatment). Sequential plasma samples collected from pre-infection, at the first HIV positive point, weeks 1, 2, 4, 8, 12 post-infection and every three months after that, till to over three years were analyzed. 20 HIV-negative MSM were used as controls. The stage of HIV-1 infection can be depicted as six discrete stages proposed by Fiebig^35^. Stage I-II: HIV RNA positive and ELISA negative. Stage III-IV: HIV RNA positive, ELISA positive, and Western blot negative or indeterminate. Stage V: HIV RNA positive, ELISA positive, and Western blot positive without P31 band. Stage VI: HIV RNA positive, ELISA positive, and Western blot positive with P31 band.

### Markers of HIV-1 Disease Progression

Absolute blood CD4^+^ T cell counts (cells/μL) were measured using a FACSCalibure flow cytometry (BD, Franklin Lakes, New Jersey, USA) at regular intervals during HIV-1 infection. Plasma HIV-1 RNA concentrations (copies/mL) were quantified using the COBAS AMPLCOR^TM^ HIV-1 Monitor v1.5 or COBAS Ampliprep/COBAS TaqMan 48 Analyser (Roche Diagnostic, Branchburg, New Jersey, USA), with a detection limit of 40 copies/mL of plasma. Viral load and CD4^+^ T cell count set points were defined as the average HIV-1 RNA or CD4^+^ T cell count measurements of at least three consecutive visits during the stable level stage between medians of 24 and 108 weeks post-infection.

### Luminex

Cytokine concentrations in plasma were determined by using a high-sensitivity human cytokine/Milliplex map kit (Millipore): Interleukin (IL)-1 receptor agonist (ra), IL-1β, IL-2, IL-4, IL-5, IL-6, IL-7, IL-8, IL-9, IL-10, IL-12, IL-13, IL-15, IL-17, epidermal growth factor (EGF)-2, eotaxin, granulocyte colony stimulating factor (G-CSF), granulocyte-macrophage (GM)-CSF, interferon (IFN)-γ, IFN-α2, IFN-gamma-induced protein (IP)-10, monocyte chemotactic protein (MCP)-1, macrophage inflammatory protein (MIP)-1α, MIP-1β, TNF-α and vascular endothelial growth factor (VEGF). Each sample was assayed in duplicate, and cytokine standards supplied by the manufacturer were run on each plate. Data were acquired using a Luminex-100 system and analyzed using Bio-Plex Manager software, v4.1 (Bio-Rad). Cytokine concentrations below the lower limits of detection were reported as the midpoint between the lowest concentration for each cytokine measured and zero.

### Statistical Analysis

Non-parametric Mann-Whitney *U* tests were used to compare the median plasma cytokine concentrations of the two disease progression groups. *P*-values < 0.05 were considered statistically significant. The correlation among plasma cytokine concentrations for healthy subjects and HIV-1-infected individuals were determined using Spearman correlation coefficients. Correlation matrices were displayed as schematic correlograms^36^. Due to the wide range of each cytokine measurement, fold change of the cytokine level over its reference level, which was determined as the median cytokine of 20 HIV-negative MSM, was used for the following dynamic analysis. The dynamics of the plasma cytokines were fitted on the change folds along the time line by locally weighted scatterplot smoothing (LOWESS) with bandwidth (the most important parameter) of 0.3 determined through trial and error. The points of the first and the second peak on the smoothing fitted curve were determined visually, and the x-axis and y-axis coordinates of the point were regarded as the duration and magnitude of cytokine elevation for that peak, respectively. All statistical analyses were conducted in Stata/SE 12 and open source procedure R 3.2.

## Additional Information

**How to cite this article**: Huang, X. *et al*. Cytokine cascade and networks among MSM HIV seroconverters: implications for early immunotherapy. *Sci. Rep*. **6**, 36234; doi: 10.1038/srep36234 (2016).

**Publisher’s note**: Springer Nature remains neutral with regard to jurisdictional claims in published maps and institutional affiliations.

## Supplementary Material

Supplementary Information

## Figures and Tables

**Table 1:** Baseline characteristics of the HIV-infected participants.

	RDP group (n = 10)	SDP group (n = 10)	*p* value
Age (years)	26 (24.5–35)	26.5 (24–30.75)	0.786
Estimated infection day at seroconversion (days)	30 (25.75–34.25)	30 (17.75–42.25)	0.846
Follow-up (days)	414 (273.5–486.25)	612 (554–803.75)	0.023
Fiebig staging			
I-II	0 (0–30.85)	20 (2.52–55.61)	0.211
III-IV	40 (12.16–73.76)	50 (18.71–81.29)	
V-VI	60 (26.24–87.84)	30 (6.67–65.25)	
Subtype			
AE	70 (34.75–3.33)	80 (44.39–97.48)	0.356
BC	0 (0–30.85)	10 (0.25–44.5)	
B	30 (6.67–65.25)	10 (0.25–44.5)	
Acute symptoms	70 (34.75–93.33)	70 (34.75–93.33)	1.000
Active syphilis at seroconversion	60 (26.24–87.84)	30 (6.67–65.25)	0.178
HBV co-infection	0	0	
HCV co-infection	0	0	
Initial CD4^+^ T cell counts (cells/μL)	350 (242–526.75)	665 (591–801.75)	<0.001
CD4^+^ setpoint (cells/μL)	288.2 (147.2–351.3)	653.6 (555.7–848.0)	<0.001
Peak viral load (Log copies/mL)	5.14 (4.38–5.67)	4.32 (3.68–5.02)	0.046
Plasma viral load setpoint (Log copies/mL)	4.94 (4.35–5.5)	3.47 (2.77–4.54)	0.046

Data are % (95% CI) or median (IQR), unless otherwise indicated.

RDP: Rapid Disease Progressor; SDP: Slow Disease Progressor.

**Table 2:** Plasma cytokine concentrations of the HIV-infected participants [Median (IQR; pg/mL)].

Cytokine	HIV-negative MSM (n = 20)	0−180 days post-infection			0.5−3 years post-infection		
		RDP group (n = 10)	SDP group (n = 10)	*p* value^*^	RDP group (n = 10)	SDP group (n = 10)	*p* value*
Eotaxin (×10^2^)	2.4 (1.5, 3.5)	2.5 (1.7, 3.8)	2.9 (1.8, 4.1)	0.562	2.6 (1.9, 4.1)	3.1 (1.8, 4.7)	0.473
FGF-2	21.33 (13.5, 33.8)	71.3 (25.5, 157.5)	36.0 (14.3, 76.0)	0.004	71.5 (34.6, 133.1)	48.8 (23.0, 99.8)	0.004
G-CSF	27.3 (17.3, 46.0)	70.8 (29.5, 128.5)	42.5 (21.5, 73.0)	0.051	72.5 (37.3, 129.8)	58.5 (29.0, 96.6)	0.067
GM-CSF	23.8 (18.8, 35.5)	79.8 (31.5, 159.5)	32.5 (13.5, 60.5)	<0.001	67.5 (32.9, 135.1)	44.5 (18.1, 78.9)	<0.001
IFN-α2	8.0 (4.0, 12.0)	36.0 (8.0, 62.3)	7.5 (4.0, 19.0)	<0.001	29.0 (10.6, 51.1)	13.0 (4.1, 30.7)	<0.001
IFN-γ	17.8 (14.0, 22.3)	41.5 (15.8, 83.8)	20.0 (11.5, 36.0)	0.024	40.5 (17.5, 85.1)	30.0 (14.8, 58.6)	0.056
IL-1β	11.0 (−4.5, 31.8)	87.1 (31.0, 174.1)	45.9 (−6.7, 97.5)	0.014	88.7 (30.4, 177.6)	58.2 (16.6, 155.4)	0.059
IL-1ra	7.8 (0.8, 13.8)	36.0 (9.5, 65.5)	9.8 (−3.0, 28.0)	0.006	34.5 (9.33, 57.3)	17.0 (1.1, 45.5)	0.046
IL-2	20.8 (13.3, 42.0)	61.3 (25.5, 104.0)	29.5 (12.4, 61.9)	0.006	59.5 (27.9, 103.3)	40.0 (19.0, 76.0)	0.011
IL-4	−47.5 (−71.5, 10.0)	−3.8 (−10.9, 2.6)	−9.0 (−15.2, −3.2)	0.028	44.0 (−11.3, 1.3)	−7.2 (−14.5, −0.2)	0.181
IL-5	9.0 (4.2, 17.7)	−3.8 (−10.9, 2.6)	15.5 (0.0, 40.3)	<0.001	36.5 (8.5, 62.8)	24.5 (5.5, 56.3)	0.275
IL-6	4.0 (−2.5, 15.0)	72.7 (8.6, 212.5)	25.8 (2.3.65.0)	0.016	71.1 (22.3, 164.5)	35.9 (8.6, 85.9)	0.002
IL-7	11.8 (−1.0, 18.8)	50.8 (15.2, 96.6)	27.1 (2.1, 65.3)	0.108	61.5 (23.1, 106.9)	47.0 (13.7, 101.0)	0.178
IL-8	63.0 (33.0, 77.8)	82.3 (39.0, 123.5)	75.0 (41.1, 112.8)	0.817	83.0 (41.3, 129.0)	81.3 (52.3, 120.3)	0.689
IL-9	−2.0 (−8.0, 7.5)	34.3 (−2.0, 61.0)	6.0 (−6.5, 3.8)	0.034	33.0 (3.8, 59.1)	14.0 (−2.5, 44.5)	0.078
IL-10	21.5 (10.0, 30.3)	44.0 (20.3, 84.5)	18.3 (6.0, 338.5)	0.003	47.0 (20.1, 84.0)	25.0 (10.9, 52.6)	0.002
IL-12	16.8 (8.5, 20.3)	62.8 (15.5, 121.0)	24.0 (1.5, 47.0)	0.002	67.5 (26.2, 111.6)	35.5 (11.5, 66.4)	<0.001
IL-13	5.3 (−1.8, 16.0)	116.1 (9.5, 212.6)	41.7 (−1.2, 97.6)	0.054	11.6 (31.0, 215.3)	66.2 (8.0, 166.1)	0.052
IL-15	5.7 (0.6, 12.2)	37.3 (4.8, 91.5)	6.5 (−1.5, 38.3)	0.009	34.8 (6.6, 77.1)	16.2 (0.7, 43.0)	0.011
IL-17	24.3 (18.8, 33.5)	50.0 (20.5, 84.5)	27.0 (11.6, 55.1)	0.094	52.0 (24.3, 94.8)	42.5 (17.0, 81.0)	0.166
IP-10 (×10^2^)	35.2 (20.4, 56.5)	45.0 (28.3, 71.3)	23.7 (12.4, 41.0)	<0.001	41.9 (28.2, 68.0)	23.5 (11.1, 36.9)	<0.001
MCP-1 (×10^2^)	21.0 (10.0, 25.2)	13.5 (7.8, 21.0)	16.6(13.3, 21.7)	0.118	15.0 (9.8, 21.0)	16.7 (12.2, 21.5)	0.256
MIP-1α	−0.3 (−6.5, 18.5)	34.5 (5.5, 57.0)	16.0 (2.0, 31.0)	0.102	33.5 (10.1, 68.3)	18.0 (5.3, 50.8)	0.102
MIP-1β	47.5 (31.3, 59.5)	87.3 (53.5, 172.5)	56.5 (24.5, 97.5)	0.051	85.5 (53.0, 164.0)	73.5 (43.5, 135.4)	0.183
TNF-α	79.2 (52.0, 135.3)	125.8 (91.5, 186.0)	91.0 (59.0, 128.3)	0.007	123.3 (85.0, 186.3)	99.3 (69.9, 140.0)	0.006
VEGF	2.8 (−1.0, 7.3)	13.8 (4.8, 45.8)	1.9 (−6.0, 7.4)	<0.001	22.0 (4.8, 51.1)	5.7 (−1.0, 20.5)	<0.001

^*^P-value < 0.001 was considered statistically significant after Bonferroni correction.

**Table 3:** Peak values of plasma cytokines and estimated infection date in RDP group and SDP group.

Function	Cytokine	RDP group		SDP group	
		Days	Change Folds	Days	Change Folds
Inflammatory	IL-1β	57	9.64	82	5.33
	IL-6	82	5.33	77	2.44
	IL-12	57	5.77	81	3.13
	TNF-α	30	2.12	NA	NA
	IFN-α2	57	5.47	81	2.91
Chemokines	IL-8	30	1.88	82	1.81
	Eotaxin	NA	NA	NA	NA
	IP-10	30	3.26	30	1.20
	MCP-1	30	1.83	NA	NA
	MIP-1α	33	2.63	81	2.32
	MIP-1β	57	2.89	81	1.93
Anti-inflammatory	IL-1ra	33	9.06	81	7.27
	IL-10	33	3.02	81	2.08
Growth Factor	VEGF	93	14.93	71	3.73
	FGF-2	62	9.00	81	3.70
Hematopoietic	IL-7	57	2.26	82	1.90
	G-CSF	33	3.10	81	2.66
	GM-CSF	61	8.20	81	2.55
Adaptive	IFN-γ	30	13.77	81	2.51
	IL-2	61	6.28	81	3.29
	IL-4	33	5.09	54	5.21
	IL-5	36	5.99	77	6.11
	IL-9	59	5.09	81	4.54
	IL-13	105	18.38	33	9.30
	IL-15	48	18.75	82	7.81
	IL-17	33	2.71	81	2.46
Average		49.6*	6.7^#^	74.9	3.7

NA means there was no obvious peak observed.

RDP: Rapid Disease Progressor; SDP: Slow Disease Progressor.

^*^P-value < 0.001 compared with SDP group.

^#^*P*-value = 0.012 compared with SDP group.

## References

[b1] Ravimohan S. . Immunological profiling of tuberculosis-associated immune reconstitution inflammatory syndrome and non-immune reconstitution inflammatory syndrome death in HIV-infected adults with pulmonary tuberculosis starting antiretroviral therapy: a prospective observational cohort study. The Lancet. Infectious diseases 15, 429–438, doi: 10.1016/S1473-3099(15)70008-3 (2015). 25672566 PMC4624391

[b2] Stacey A. R. . Induction of a striking systemic cytokine cascade prior to peak viremia in acute human immunodeficiency virus type 1 infection, in contrast to more modest and delayed responses in acute hepatitis B and C virus infections. Journal of virology 83, 3719–3733, doi: 10.1128/JVI.01844-08 (2009). 19176632 PMC2663284

[b3] Norris P. J. . Elevations in IL-10, TNF-alpha, and IFN-gamma from the earliest point of HIV Type 1 infection. AIDS Res Hum Retroviruses 22, 757–762, doi: 10.1089/aid.2006.22.757 (2006). 16910831 PMC2431151

[b4] Roe B. . Elevated serum levels of interferon- gamma -inducible protein-10 in patients coinfected with hepatitis C virus and HIV. The Journal of infectious diseases 196, 1053–1057, doi: 10.1086/520935 (2007). 17763328

[b5] Jiao Y. . Plasma IP-10 is associated with rapid disease progression in early HIV-1 infection. Viral immunology 25, 333–337, doi: 10.1089/vim.2012.0011 (2012). 22788418

[b6] Sinicco A. . Cytokine network and acute primary HIV-1 infection. Aids 7, 1167–1172 (1993). 8216972 10.1097/00002030-199309000-00003

[b7] Borrow P., Shattock R. J., Vyakarnam A. & Group E. W. Innate immunity against HIV: a priority target for HIV prevention research. Retrovirology 7, 84, doi: 10.1186/1742-4690-7-84 (2010). 20937128 PMC2964587

[b8] Barcellini W. . Cytokines and soluble receptor changes in the transition from primary to early chronic HIV type 1 infection. AIDS Res Hum Retroviruses 12, 325–331, doi: 10.1089/aid.1996.12.325 (1996). 8906993

[b9] Catalfamo M., Le Saout C. & Lane H. C. The role of cytokines in the pathogenesis and treatment of HIV infection. Cytokine Growth Factor Rev 23, 207–214, doi: 10.1016/j.cytogfr.2012.05.007 (2012). 22738931 PMC3726258

[b10] Vanpouille C. . Distinct cytokine/chemokine network in semen and blood characterize different stages of HIV infection. Aids 30, 193–201, doi: 10.1097/QAD.0000000000000964 (2016). 26558730 PMC4862605

[b11] Katsikis P. D., Mueller Y. M. & Villinger F. The cytokine network of acute HIV infection: a promising target for vaccines and therapy to reduce viral set-point? PLoS pathogens 7, e1002055, doi: 10.1371/journal.ppat.1002055 (2011). 21852945 PMC3154847

[b12] Stifter S. A. & Feng C. G. Interfering with immunity: detrimental role of type I IFNs during infection. J Immunol 194, 2455–2465, doi: 10.4049/jimmunol.1402794 (2015). 25747907

[b13] Chang J. J. & Altfeld M. Innate immune activation in primary HIV-1 infection. J Infect Dis 202 Suppl 2, S297–S301, doi: 10.1086/655657 (2010). 20846036 PMC2945608

[b14] Davidson S., Maini M. K. & Wack A. Disease-promoting effects of type I interferons in viral, bacterial, and coinfections. J Interferon Cytokine Res 35, 252–264, doi: 10.1089/jir.2014.0227 (2015). 25714109 PMC4389918

[b15] Lisco A. . HIV-1 imposes rigidity on blood and semen cytokine networks. American journal of reproductive immunology 68, 515–521, doi: 10.1111/aji.12015 (2012). 23006048 PMC3493688

[b16] Shebl F. M., Yu K., Landgren O., Goedert J. J. & Rabkin C. S. Increased levels of circulating cytokines with HIV-related immunosuppression. AIDS Res Hum Retroviruses 28, 809–815, doi: 10.1089/AID.2011.0144 (2012). 21962239 PMC3399552

[b17] Roberts L. . Plasma cytokine levels during acute HIV-1 infection predict HIV disease progression. AIDS 24, 819–831, doi: 10.1097/QAD.0b013e3283367836 (2010). 20224308 PMC3001189

[b18] Campillo-Gimenez L. . AIDS progression is associated with the emergence of IL-17-producing cells early after simian immunodeficiency virus infection. J Immunol 184, 984–992, doi: 10.4049/jimmunol.0902316 (2010). 20018630

[b19] Deeks S. G., Tracy R. & Douek D. C. Systemic effects of inflammation on health during chronic HIV infection. Immunity 39, 633–645, doi: 10.1016/j.immuni.2013.10.001 (2013). 24138880 PMC4012895

[b20] Huang X. . Rate of CD4 decline and HIV-RNA change following HIV seroconversion in men who have sex with men: a comparison between the Beijing PRIMO and CASCADE cohorts. J Acquir Immune Defic Syndr 62, 441–446, doi: 10.1097/QAI.0b013e31827f5c9a (2013). 23221982

[b21] Jia Z. . HIV burden in men who have sex with men: a prospective cohort study 2007-2012. Sci Rep 5, 11205, doi: 10.1038/srep11205 (2015). 26135810 PMC5393284

[b22] Kornfeld C. . Antiinflammatory profiles during primary SIV infection in African green monkeys are associated with protection against AIDS. J Clin Invest 115, 1082–1091, doi: 10.1172/JCI23006 (2005). 15761496 PMC1062895

[b23] Imami N. & Herasimtschuk A. A. Multifarious immunotherapeutic approaches to cure HIV-1 infection. Hum Vaccin Immunother 11, 2287–2293, doi: 10.1080/21645515.2015.1021523 (2015). 26048144 PMC4635699

[b24] Kenway-Lynch C. S., Das A., Pan D., Lackner A. A. & Pahar B. Dynamics of cytokine/chemokine responses in intestinal CD4+ and CD8+ T Cells during Acute Simian Immunodeficiency Virus Infection. J Virol 87, 11916–11923, doi: 10.1128/JVI.01750-13 (2013). 23966391 PMC3807351

[b25] Falasca K. . Cytokine patterns correlate with liver damage in patients with chronic hepatitis B and C. Annals of clinical and laboratory science 36, 144–150 (2006). 16682509

[b26] Salto-Tellez M., Tan E. & Lim B. ARDS in SARS: cytokine mediators and treatment implications. Cytokine 29, 92–94, doi: 10.1016/j.cyto.2004.09.002 (2005). 15598444 PMC7128468

[b27] Herasimtschuk A. . Therapeutic immunisation plus cytokine and hormone therapy improves CD4 T-cell counts, restores anti-HIV-1 responses and reduces immune activation in treated chronic HIV-1 infection. Vaccine 32, 7005–7013, doi: 10.1016/j.vaccine.2014.09.072 (2014). 25454870

[b28] Roff S. R., Noon-Song E. N. & Yamamoto J. K. The Significance of Interferon-gamma in HIV-1 Pathogenesis, Therapy, and Prophylaxis. Frontiers in immunology 4, 498, doi: 10.3389/fimmu.2013.00498 (2014). 24454311 PMC3888948

[b29] Papasavvas E. . IL-13 acutely augments HIV-specific and recall responses from HIV-1-infected subjects *in vitro* by modulating monocytes. J Immunol 175, 5532–5540 (2005). 16210662 10.4049/jimmunol.175.8.5532

[b30] Reuter M. A., Pombo C. & Betts M. R. Cytokine production and dysregulation in HIV pathogenesis: lessons for development of therapeutics and vaccines. Cytokine Growth Factor Rev 23, 181–191, doi: 10.1016/j.cytogfr.2012.05.005 (2012). 22743036 PMC3582023

[b31] Vandergeeten C., Fromentin R. & Chomont N. The role of cytokines in the establishment, persistence and eradication of the HIV reservoir. Cytokine Growth Factor Rev 23, 143–149, doi: 10.1016/j.cytogfr.2012.05.001 (2012). 22743037 PMC3767481

[b32] Anderson J. A. . HIV-1 Populations in Semen Arise through Multiple Mechanisms. PLoS pathogens 6, e1001053, doi: 10.1371/journal.ppat.1001053 (2010). 20808902 PMC2924360

[b33] Ford E. S., Puronen C. E. & Sereti I. Immunopathogenesis of asymptomatic chronic HIV Infection: the calm before the storm. Current opinion in HIV and AIDS 4, 206–214, doi: 10.1097/COH.0b013e328329c68c (2009). 19532052 PMC2760925

[b34] Weintrob A. C. . Syphilis co-infection does not affect HIV disease progression. International journal of STD & AIDS 21, 57–59, doi: 10.1258/ijsa.2009.009164 (2010). 19933204

[b35] Fiebig E. W. . Dynamics of HIV viremia and antibody seroconversion in plasma donors: implications for diagnosis and staging of primary HIV infection. Aids 17, 1871–1879, doi: 10.1097/01.aids.0000076308.76477.b8 (2003). 12960819

[b36] M. F. Corrgrams: Exploratory Displays for Correlation Matrices. American Statistician 56, 316–324 (2002).

