# Supplementary material for: Cytokine cascade and networks among MSM HIV seroconverters: implications for early immunotherapy
Source: Sci Rep. 2016 Nov 10;6:36234. doi: 10.1038/srep36234 (PMC5103227; doi:10.1038/srep36234)
Supplement: Supplementary Information [file srep36234-s1.pdf]

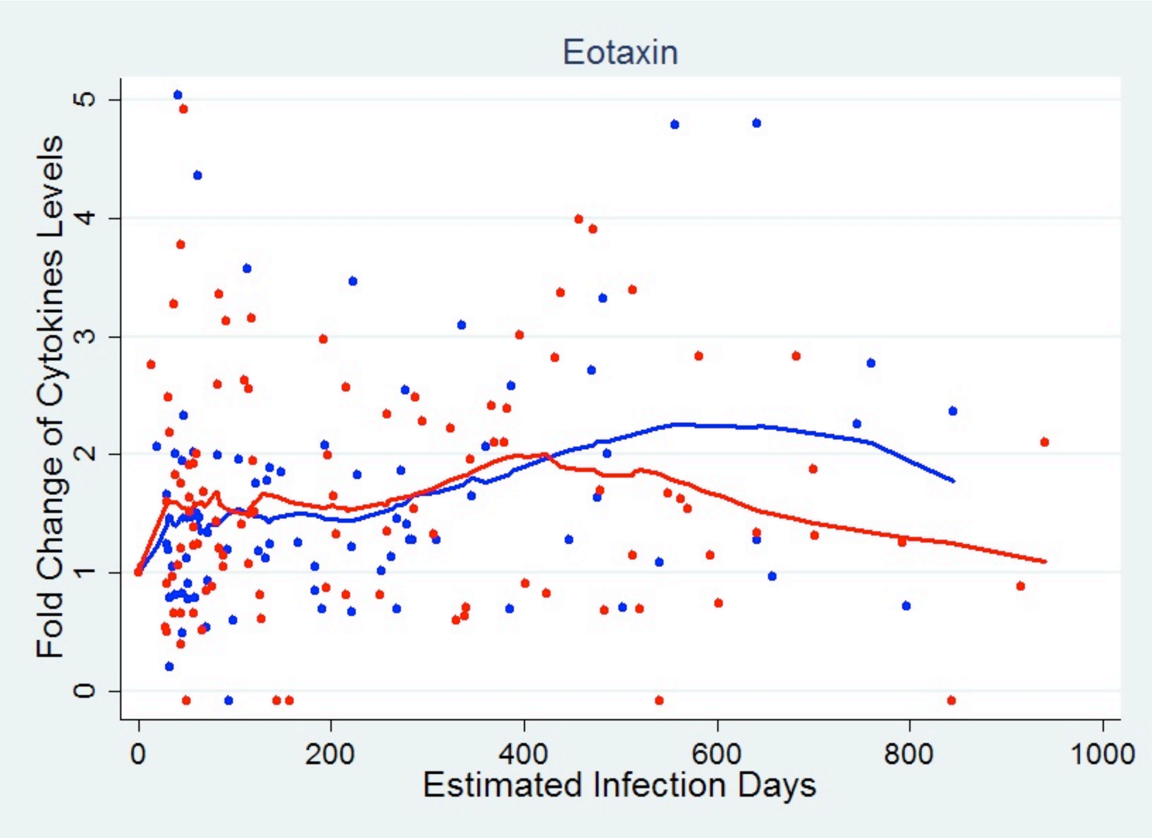
Eotaxin
Fold Change of Cytokines Levels
0
1
2
3
4
5
0
200
400
600
800
1000
Estimated Infection Days

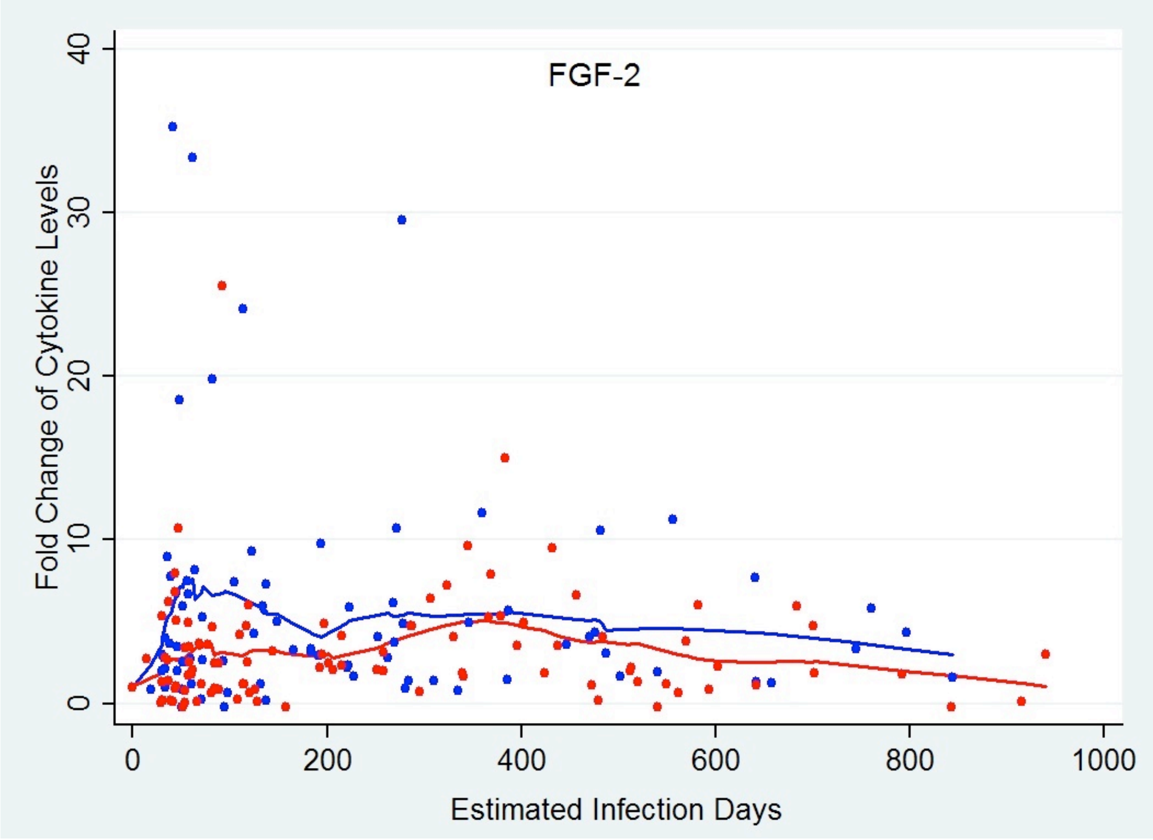
FGF-2
Fold Change of Cytokine Levels
0
10
20
30
40
0
200
400
600
800
1000
Estimated Infection Days

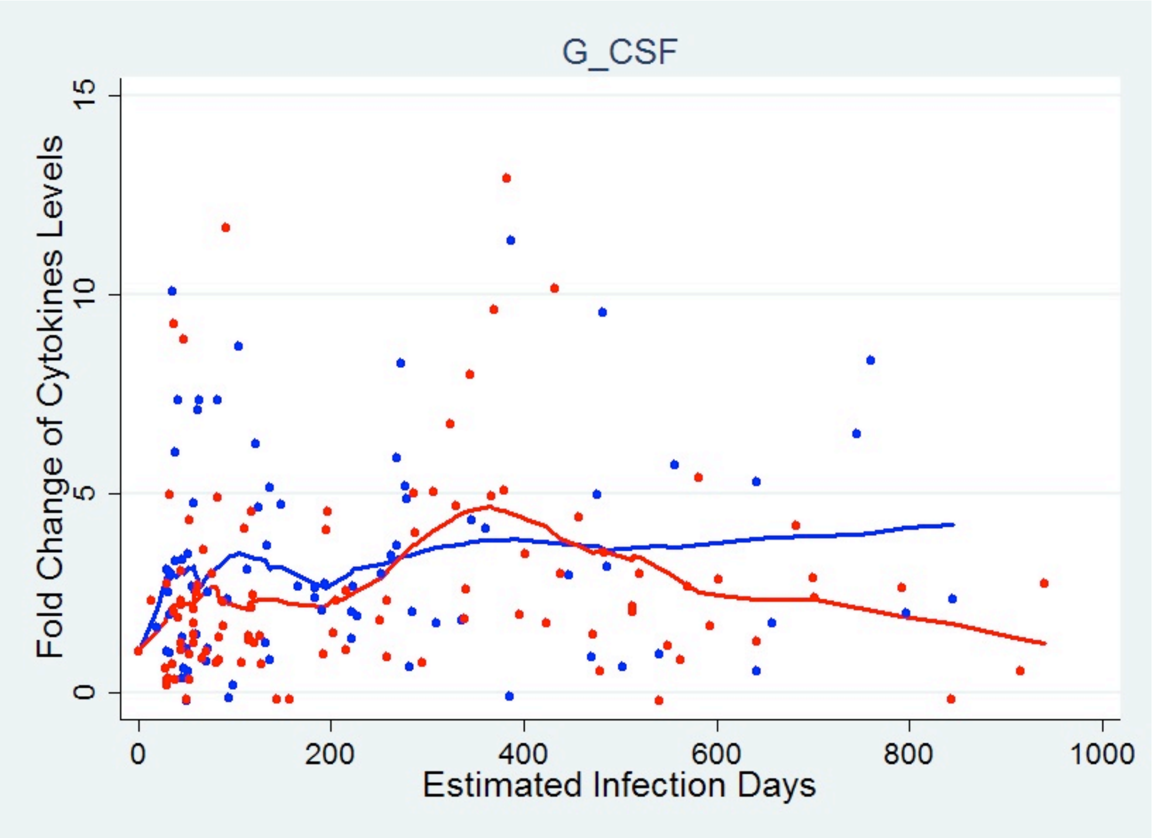
G_CSF
Fold Change of Cytokines Levels
0
5
10
15
0
200
400
600
800
1000
Estimated Infection Days

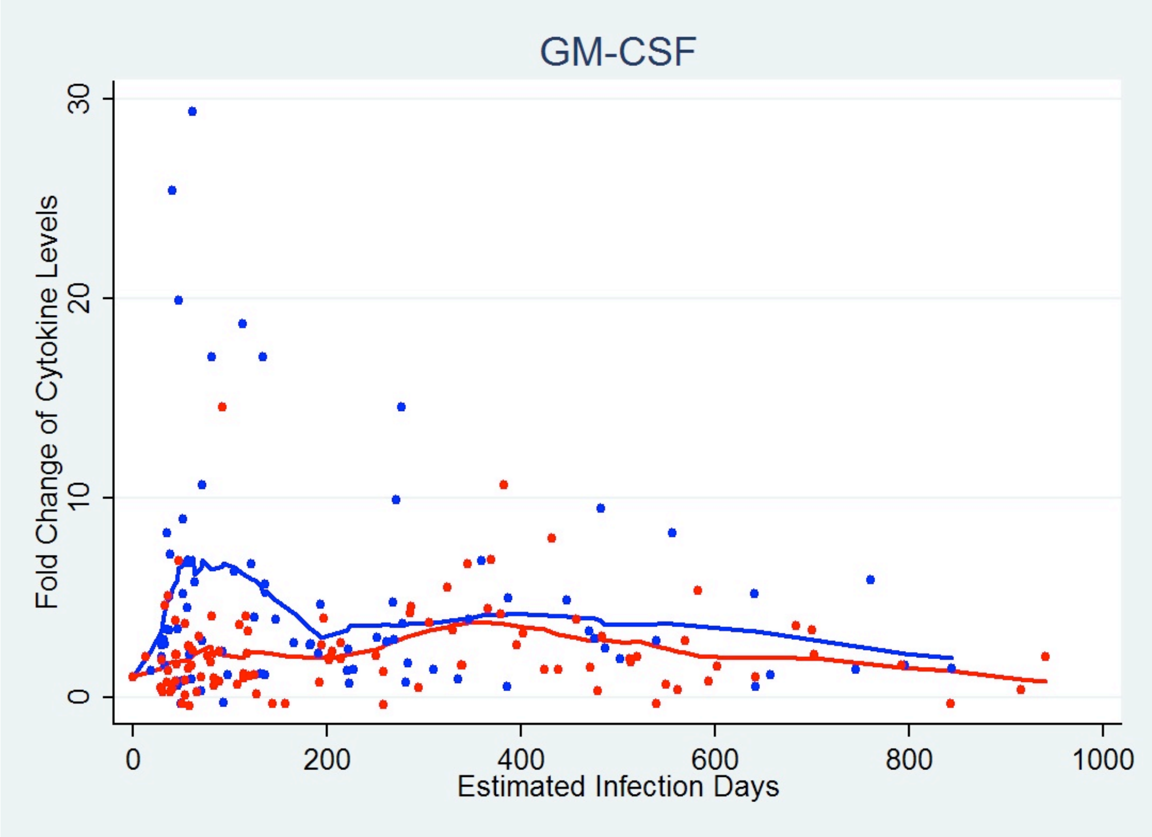
GM-CSF
Fold Change of Cytokine Levels
0
10
20
30
0
200
400
600
800
1000
Estimated Infection Days

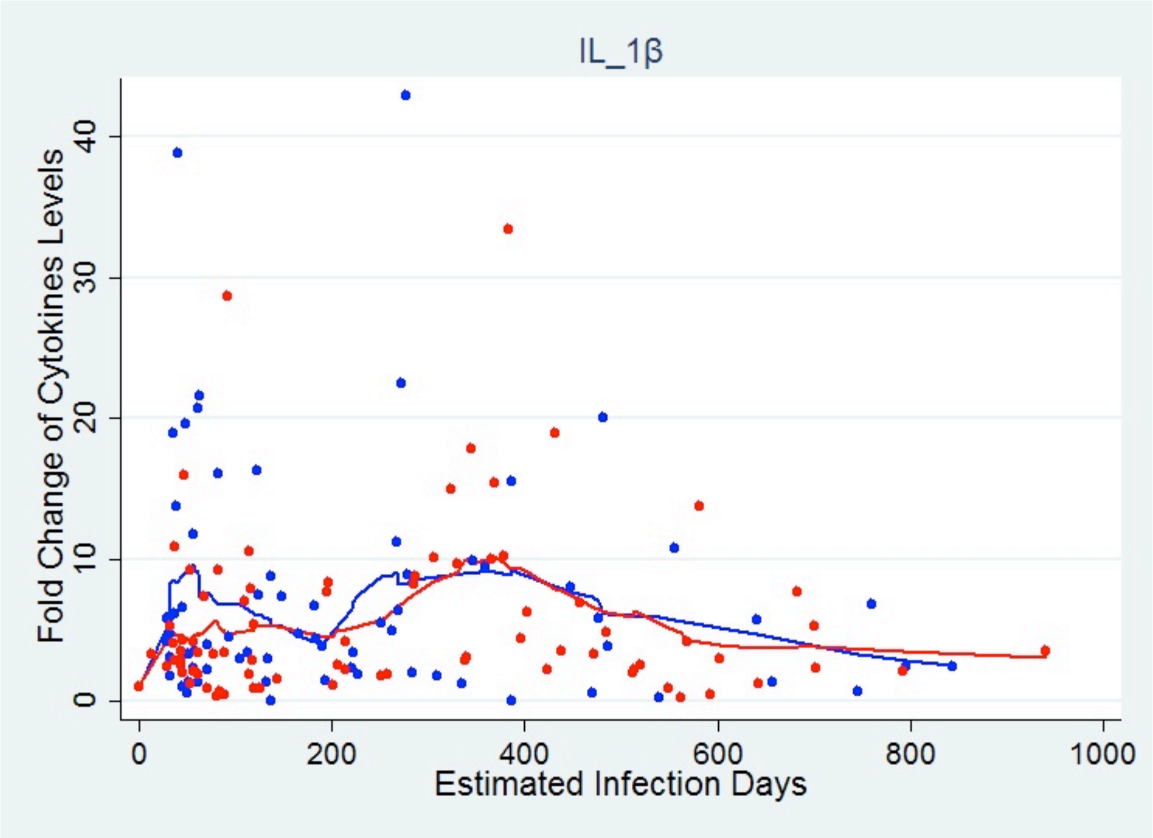
IL_1β
Fold Change of Cytokines Levels
0
10
20
30
40
0
200
400
600
800
1000
Estimated Infection Days

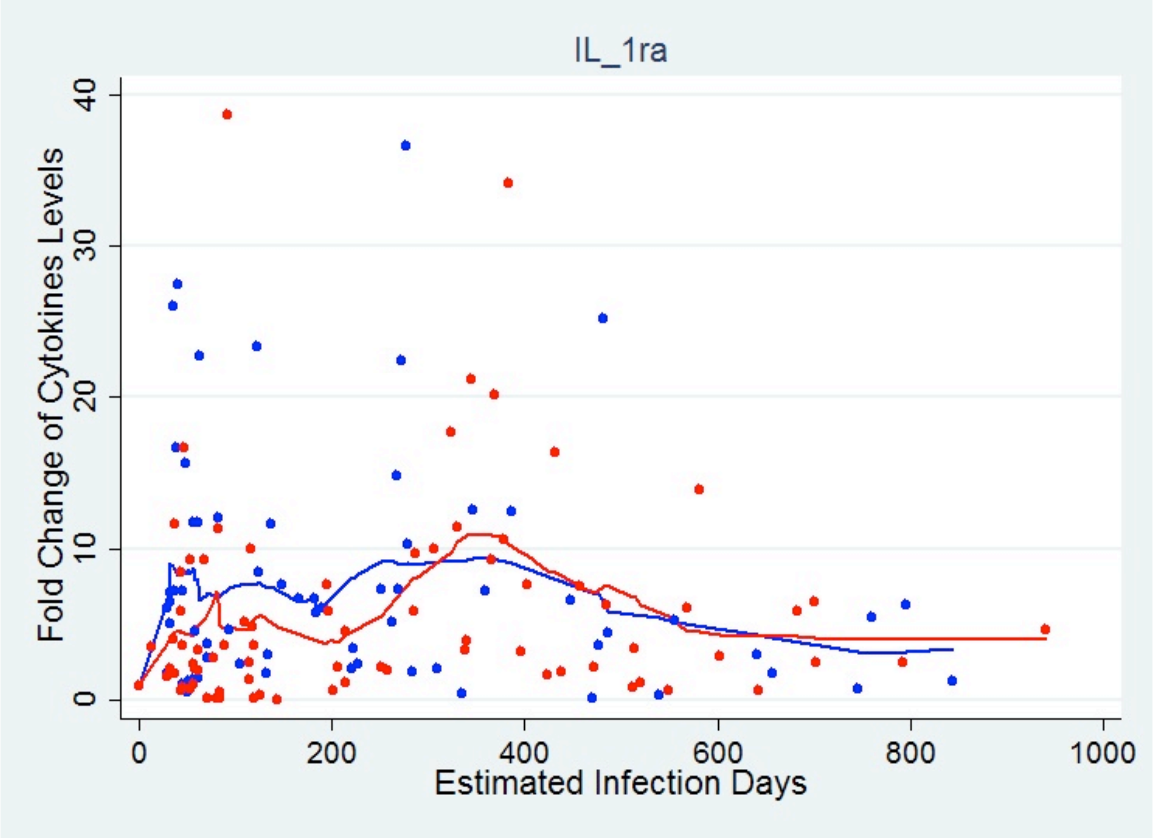
IL_1ra
Fold Change of Cytokines Levels
0
10
20
30
40
0
200
400
600
800
1000
Estimated Infection Days

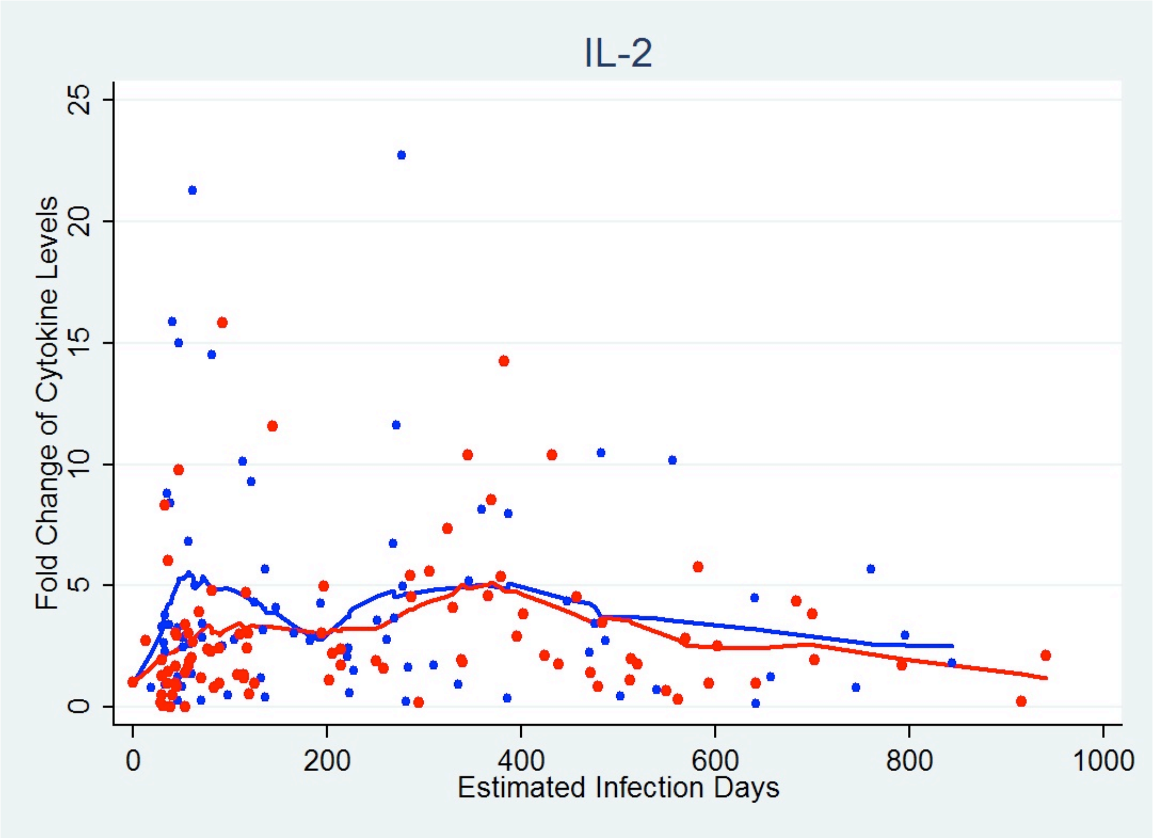
IL-2
Fold Change of Cytokine Levels
0
5
10
15
20
25
0
200
400
600
800
1000
Estimated Infection Days

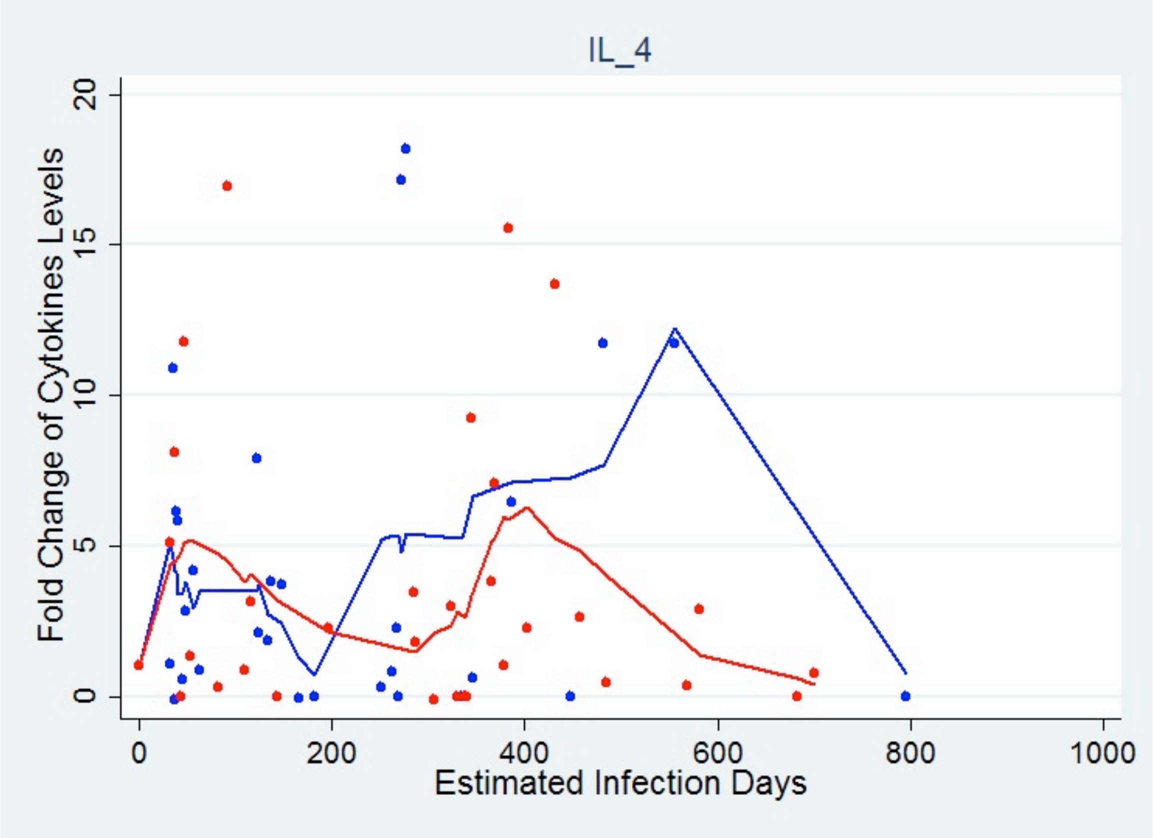
IL_4
Fold Change of Cytokines Levels
0
5
10
15
20
0
200
400
600
800
1000
Estimated Infection Days

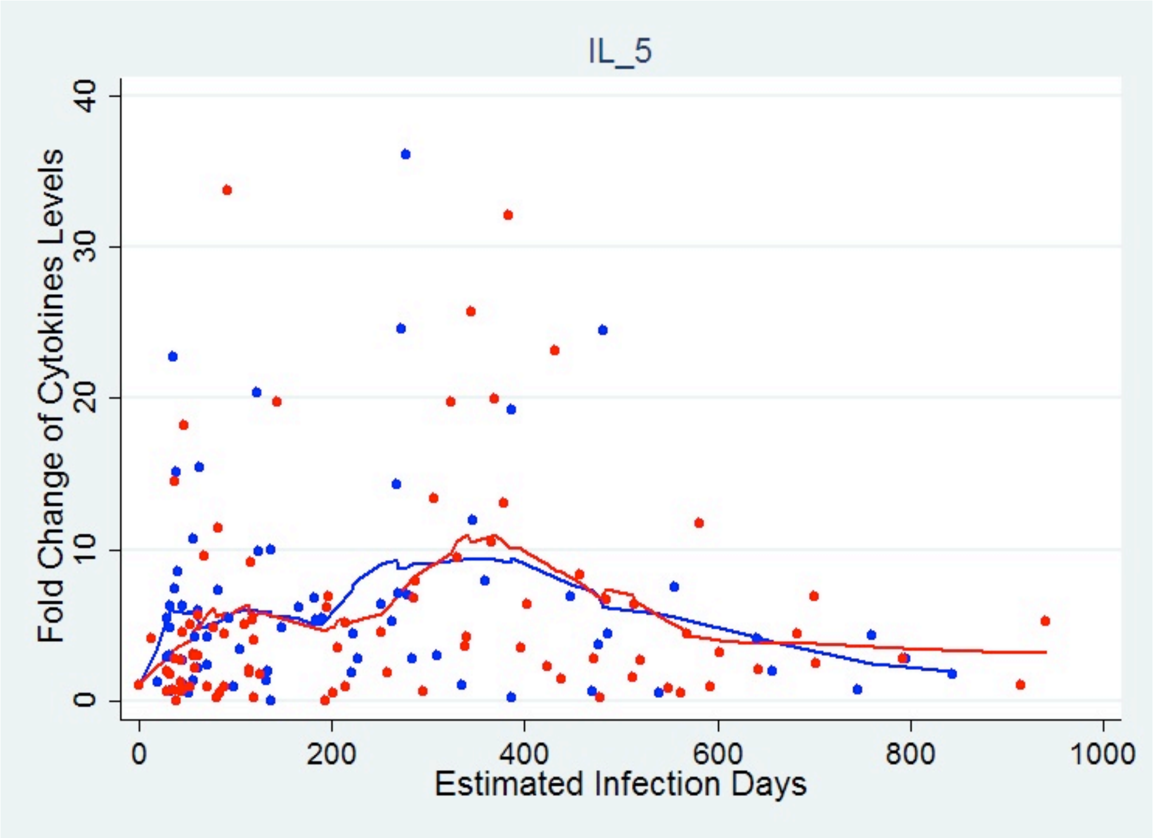
IL_5
Fold Change of Cytokines Levels
0
10
20
30
40
0
200
400
600
800
1000
Estimated Infection Days

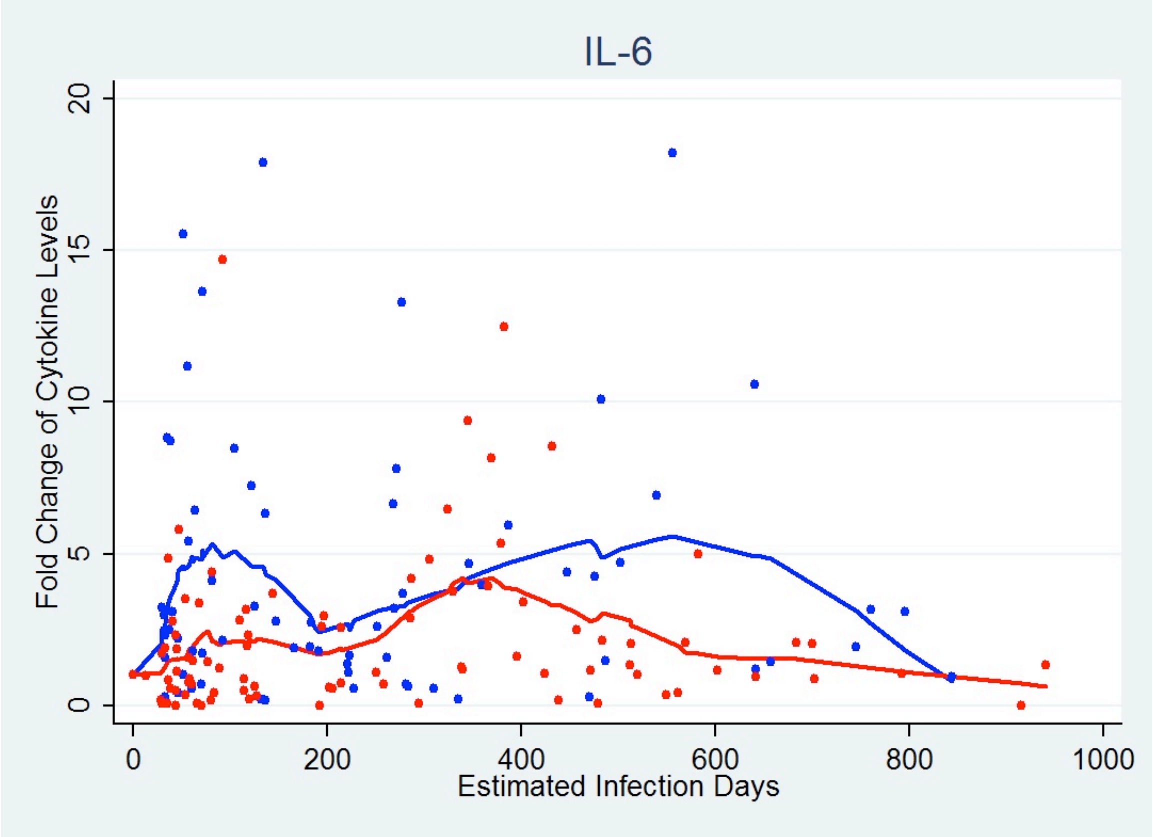
IL-6
Fold Change of Cytokine Levels
0
5
10
15
20
0
200
400
600
800
1000
Estimated Infection Days

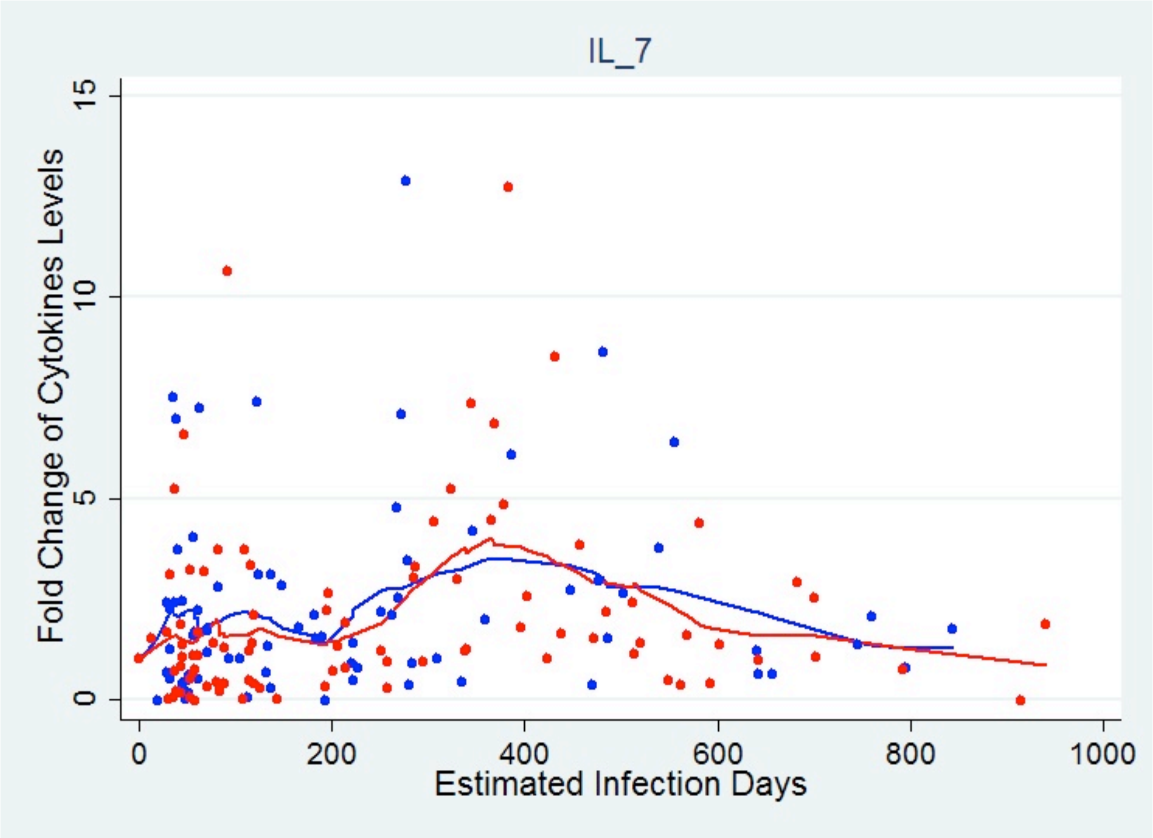
IL_7
Fold Change of Cytokines Levels
15
10
5
0
0
200
400
600
800
1000
Estimated Infection Days

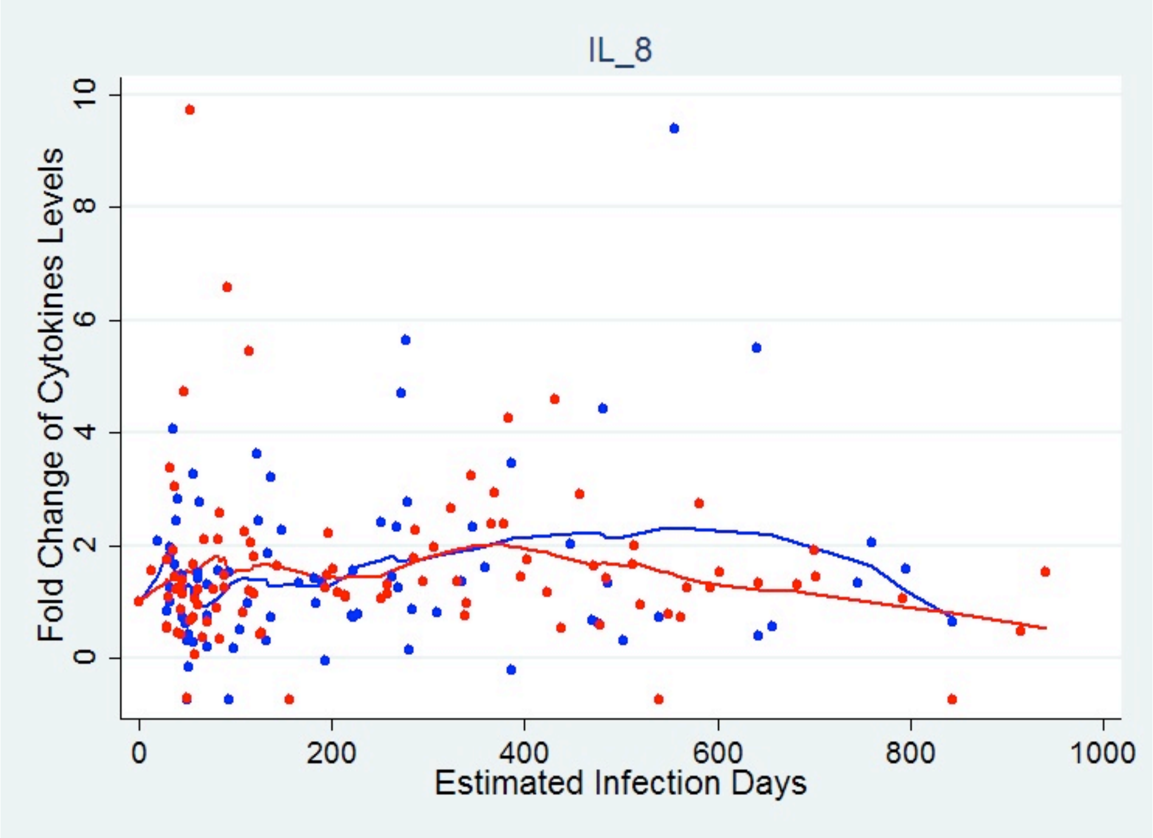
IL_8
Fold Change of Cytokines Levels
10
8
6
4
2
0
0
200
400
600
800
1000
Estimated Infection Days

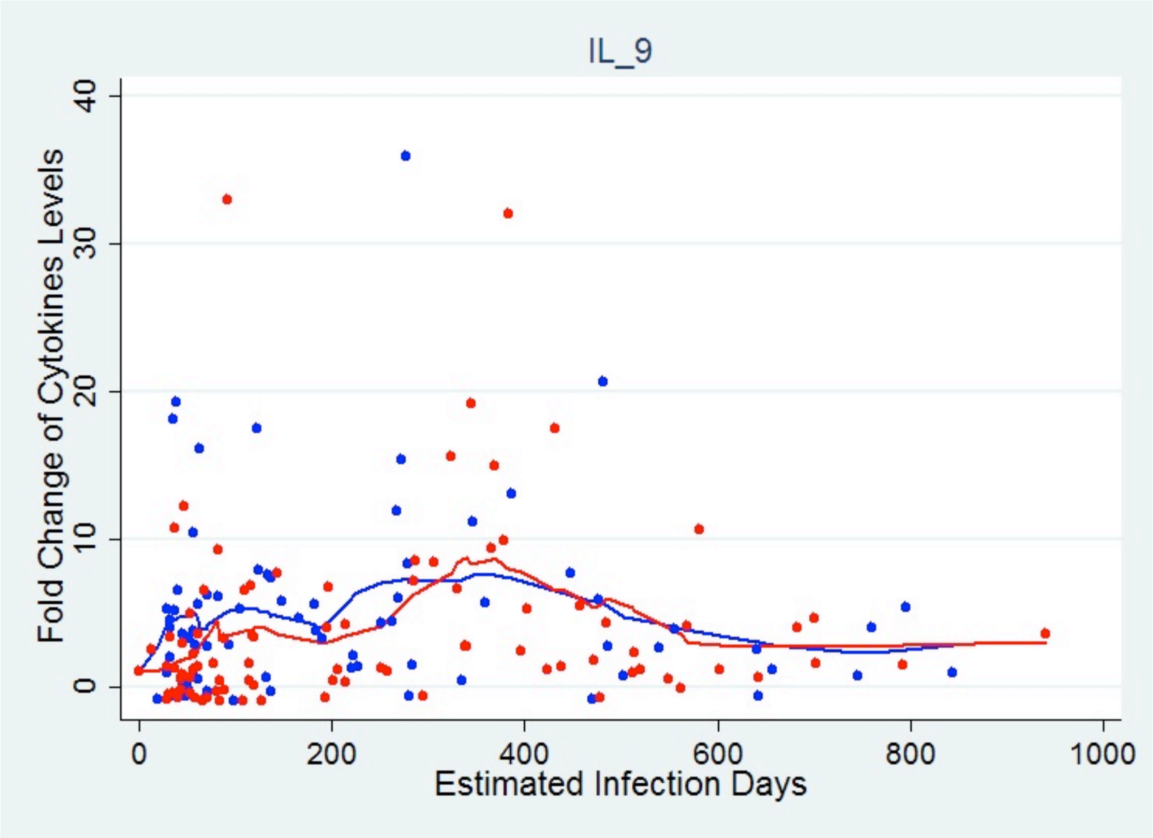
IL_9
Fold Change of Cytokines Levels
0
10
20
30
40
0
200
400
600
800
1000
Estimated Infection Days

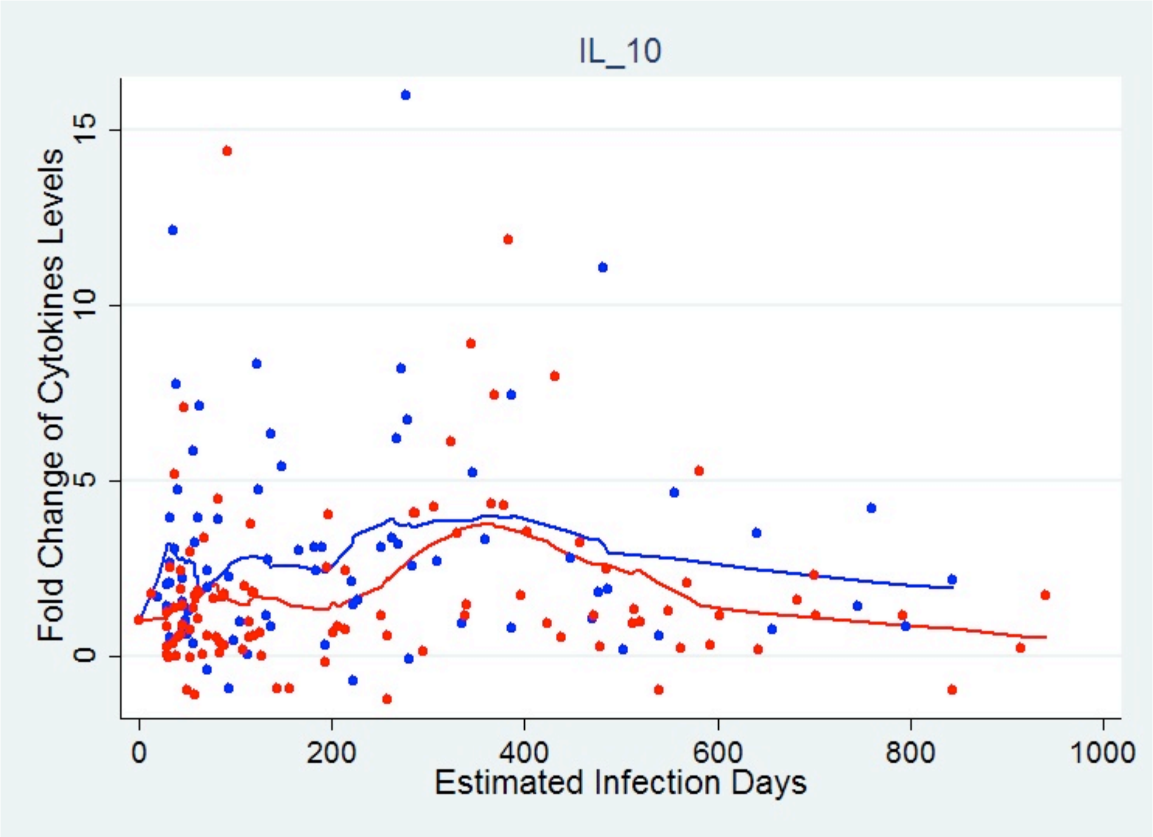
IL_10
Fold Change of Cytokines Levels
0
5
10
15
0
200
400
600
800
1000
Estimated Infection Days

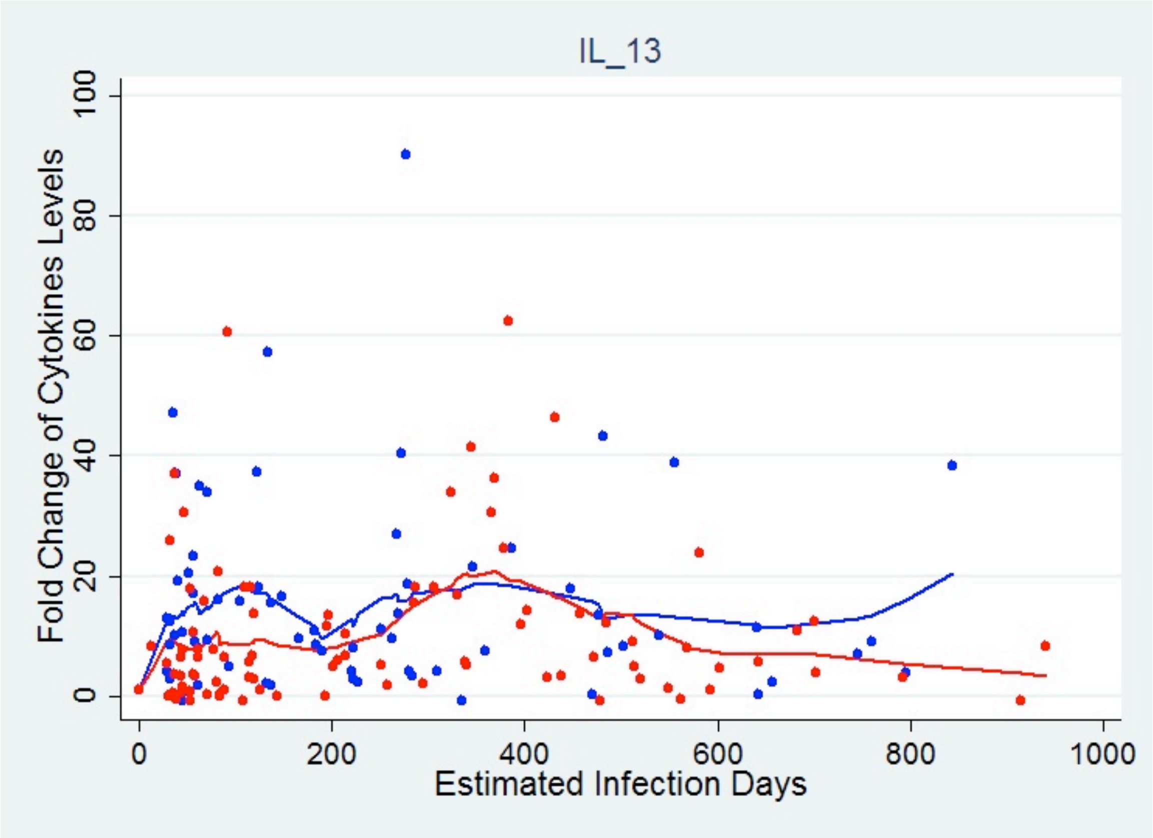
IL_13
Fold Change of Cytokines Levels
0
20
40
60
80
100
0
200
400
600
800
1000
Estimated Infection Days

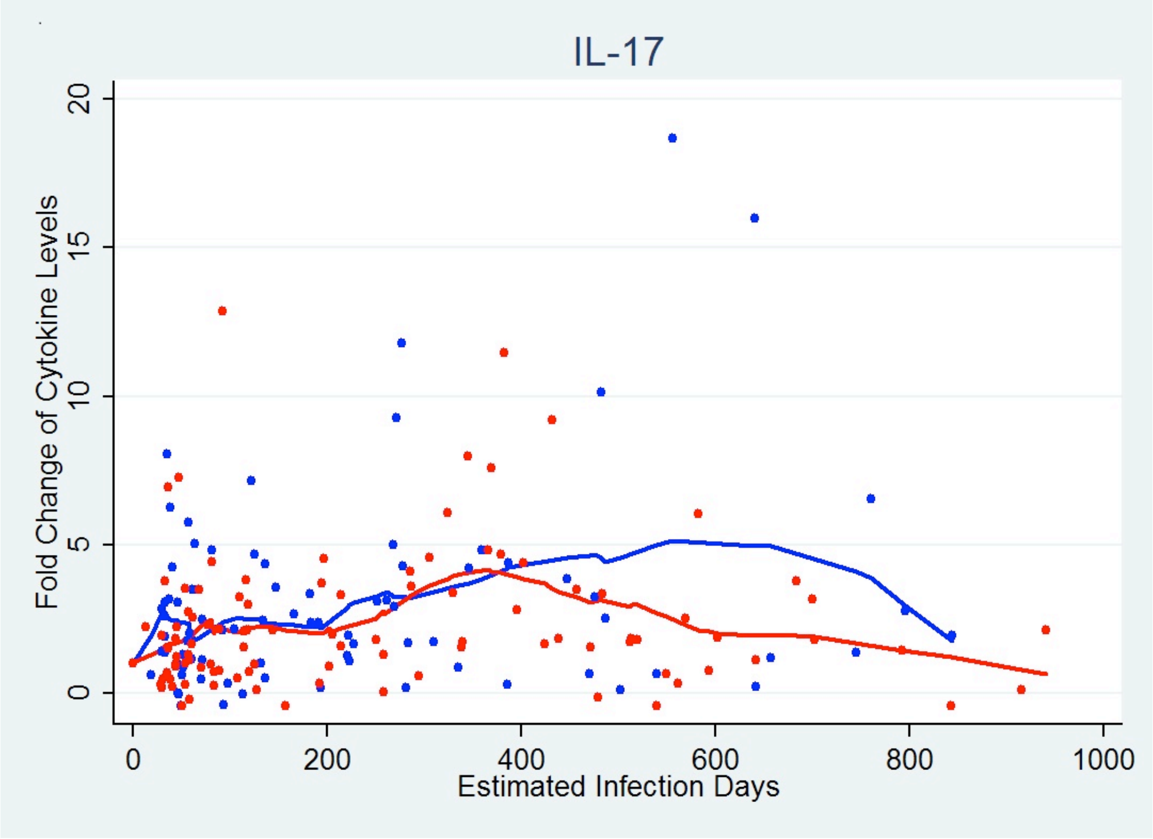
IL-17
Fold Change of Cytokine Levels
0
5
10
15
20
0
200
400
600
800
1000
Estimated Infection Days

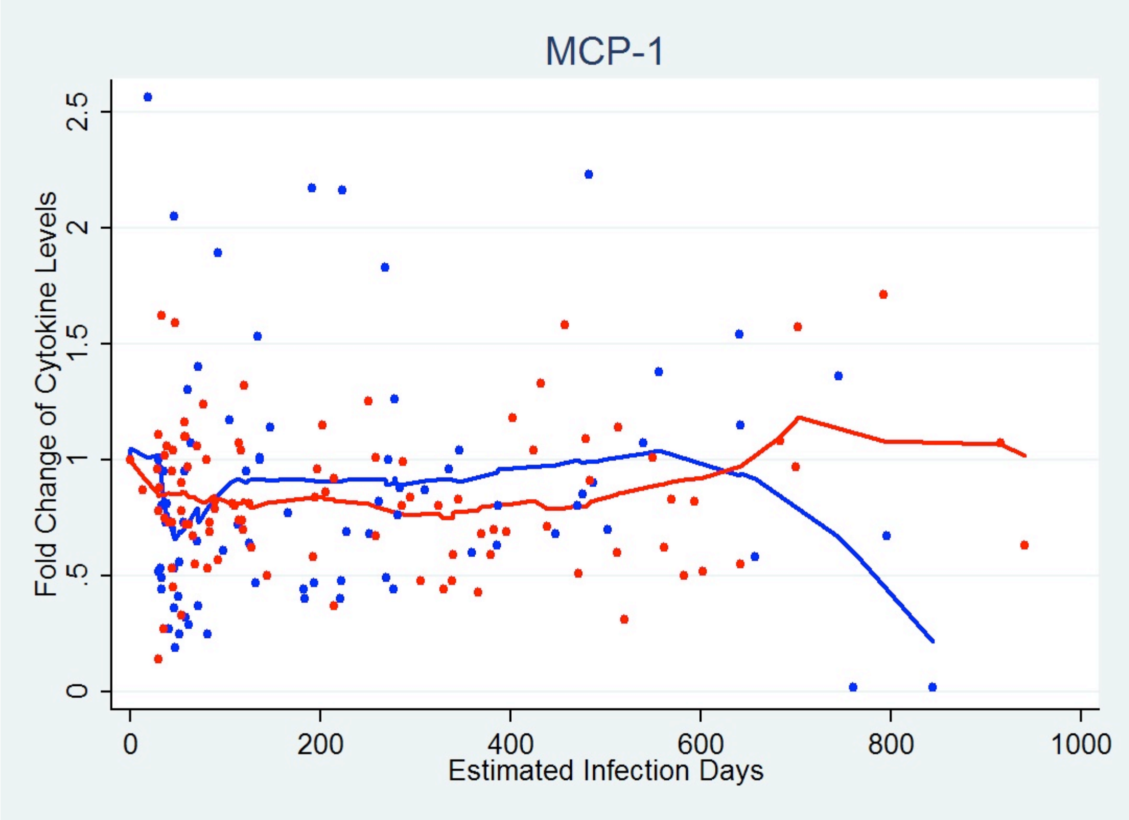
MCP-1
Fold Change of Cytokine Levels
0
.5
1
1.5
2
2.5
0
200
400
600
800
1000
Estimated Infection Days

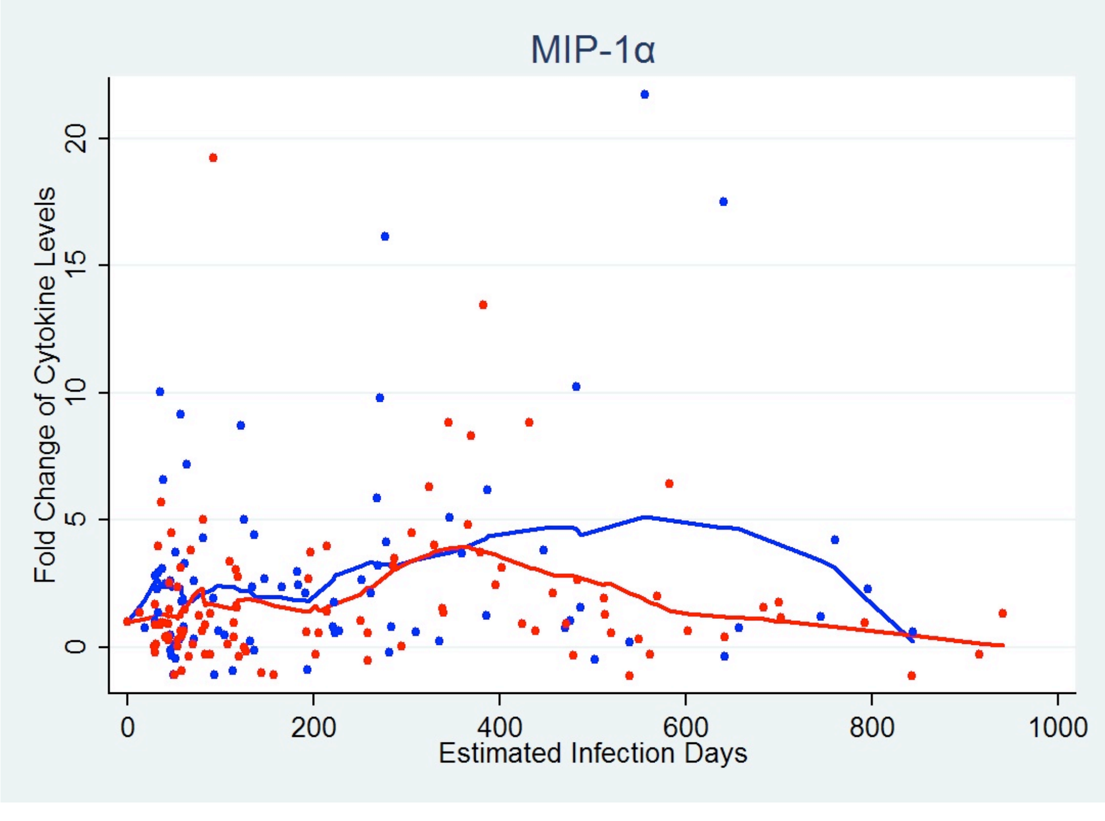
MIP-1α
Fold Change of Cytokine Levels
0
5
10
15
20
0
200
400
600
800
1000
Estimated Infection Days

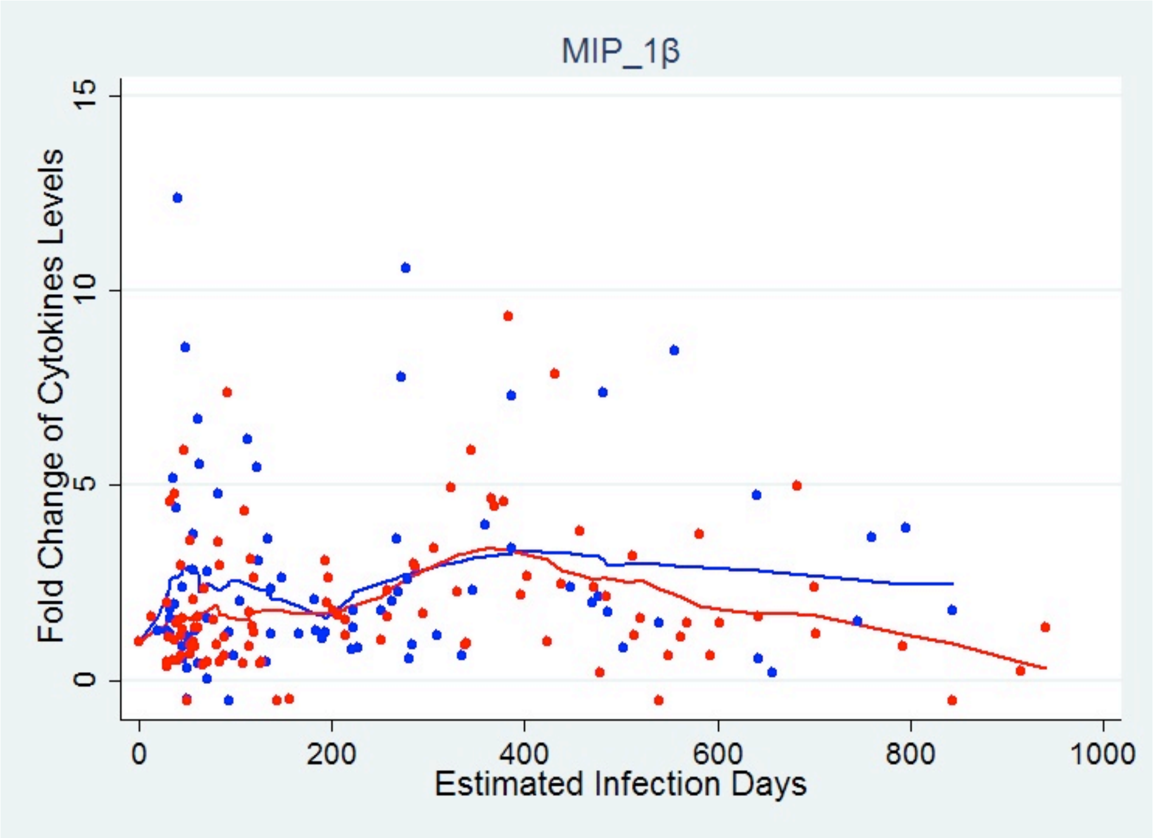
MIP_1β
Fold Change of Cytokines Levels
0
5
10
15
0
200
400
600
800
1000
Estimated Infection Days

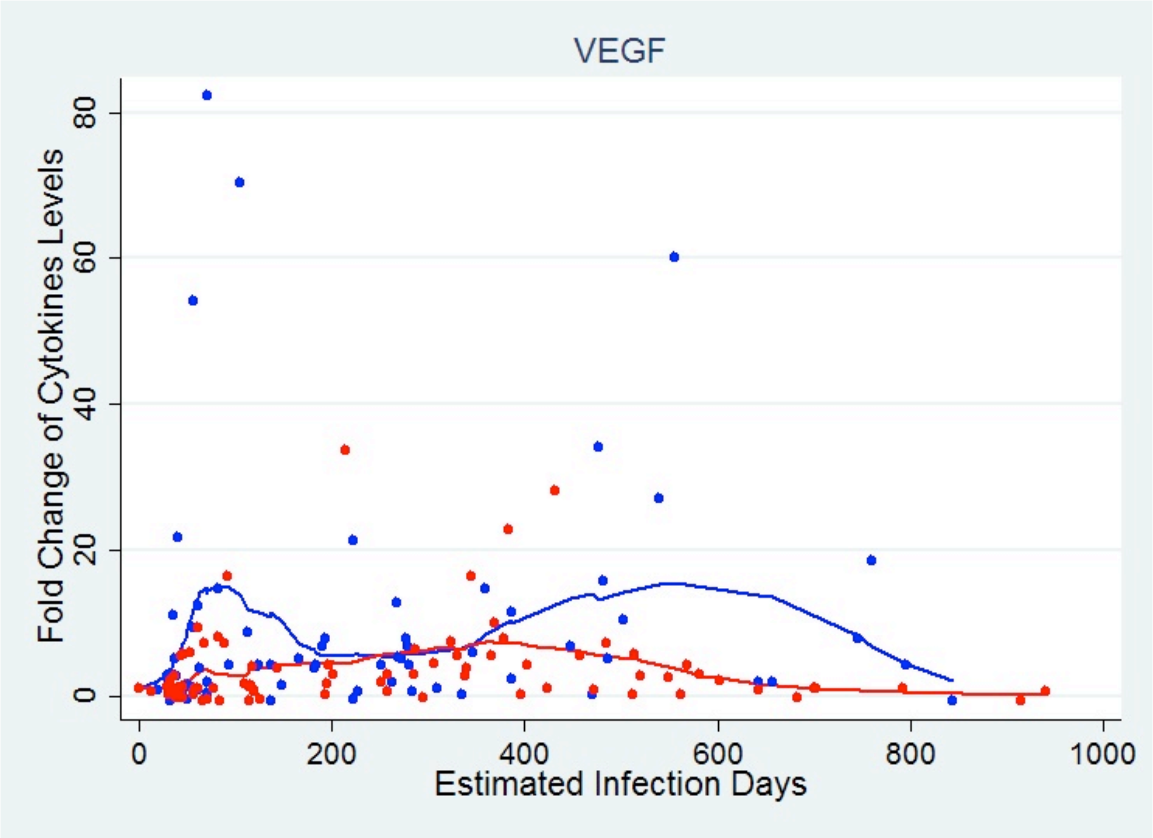
VEGF
Fold Change of Cytokines Levels
0
20
40
60
80
0
200
400
600
800
1000
Estimated Infection Days

Figure S1. The dynamic fold changes of plasma concentrations of 20 cytokines with infection dates in rapid disease progressors (RDPs) (blue dots) and slow disease progressors (SDPs) (red dots). The blue and red lines are the locally weighted scatterplot smoothing curves for RDPs and SDPs, respectively.
